# Family Support Protocol for Adolescent Internalizing Disorders: Protocol for a Pre-Post Quantitative Treatment Development Study

**DOI:** 10.2196/64332

**Published:** 2024-09-16

**Authors:** Aaron Hogue, Molly Bobek, Nicole P Porter, Alexandra MacLean, Craig E Henderson, Amanda Jensen-Doss, Gary M Diamond, Michael A Southam-Gerow, Jill Ehrenreich-May

**Affiliations:** 1 Family and Adolescent Clinical Technology & Science Partnership to End Addiction New York, NY United States; 2 Department of Psychology Sam Houston State University Huntsville, TX United States; 3 Department of Psychology University of Miami Coral Gables, FL United States; 4 Department of Psychology Ben-Gurion University of the Negev Be-er Sheva Israel; 5 Department of Psychology Virginia Commonwealth University Richmond, VA United States

**Keywords:** adolescent substance use, adolescent anxiety and depression, cooccurring disorders, adjunctive treatment, family-based interventions, usual care

## Abstract

**Background:**

Internalizing disorders (IDs), primarily depression and anxiety, are highly prevalent among adolescents receiving community-based treatment for substance use disorders (SUDs). For such clients, interventions that do not holistically address both SUDs and IDs are less effective.

**Objective:**

This pilot treatment development study aims to develop and test a modular treatment protocol for addressing cooccurring IDs among adolescents (aged 13 to 18 years) enrolled in routine care for substance use problems: Family Support Protocol for Adolescent Internalizing Disorders (Fam-AID). As an adjunctive protocol, Fam-AID will not require clinicians to markedly alter existing base practices for SUD. It will be anchored by 3 evidence-based foundations for treating cooccurring adolescent IDs: family engagement techniques, transdiagnostic individual cognitive behavioral therapy techniques, and family psychoeducation and safety planning.

**Methods:**

This quasi-experimental study will proceed in 2 stages. The pilot stage will use rapid-cycle prototyping methods in collaboration with end-user stakeholders to draft protocol delivery and fidelity guidelines adapted from existing resources, solicit provider and client input on protocol content and delivery via cognitive interviewing, and pilot prototype components on 4 to 6 cases. The second stage will be an interrupted time series study for 60 comorbid SUD+ID cases across 2 sites serving diverse adolescents: 30 will receive treatment as usual (TAU); following clinician training in the protocol, 30 new cases will receive TAU enhanced by Fam-AID. For aim 1, the focus is on evaluating the acceptability of the Fam-AID protocol through therapist and client interviews as well as assessing fidelity benchmarks using therapist- and observer-reported protocol fidelity data. For aim 2, the plan is to compare the effects of TAU only cases versus TAU+Fam-AID cases on family treatment attendance and on adolescent ID and substance use symptoms, with measurements taken at baseline and at 3-month and 6-month follow-ups.

**Results:**

Study recruitment will begin in April 2025.

**Conclusions:**

We anticipate that Fam-AID will contain 5 treatment modules that can be delivered in any sequence to meet client needs: *family engagement* of primary supports in treatment planning and services; *relational reframing* of family constraints, resiliencies, and social capital connected to the adolescent’s ID symptoms; *functional analysis* of the adolescent’s ID symptoms and related behaviors; *cognitive behavioral therapy* to address the adolescent’s ID symptoms and functional needs, featuring 3 core techniques (emotion acceptance, emotional exposure, and behavioral activation) to address negative affect and emotional dysregulation; and *family psychoeducation and safety planning* focused on education about comorbid SUD+ID and prevention of adolescent self-harm. If the abovementioned modules are found to be feasible and effective, Fam-AID will offer a set of pragmatic interventions to SUD clinicians for treating cooccurring IDs in adolescent clients.

**Trial Registration:**

ClinicalTrials.gov NCT06413979; https://www.clinicaltrials.gov/study/NCT06413979

**International Registered Report Identifier (IRRID):**

PRR1-10.2196/64332

## Introduction

### Anxiety and Depression Among Adolescents With Substance Use Disorders: Highly Prevalent and Disruptive

Anxiety (including trauma-related problems) and depression are highly prevalent among adolescents who misuse substances. National and community surveys report that adolescents with substance use disorders (SUDs) have comorbidity rates ranging between 17% and 30% for an anxiety disorder, major depressive disorder, or both [1]. Comorbidity is even higher in clinical samples. Adolescents enrolled in SUD treatment have cooccurrence rates ranging from 22% to 26% for anxiety disorders and 24% to 50% for depression disorders, along with an alarming range of 55% to 70% for clinically elevated symptoms of either disorder [2,3]. Anxiety and depression diagnoses themselves cooccur at such high rates in adolescents that prevailing clinical taxonomies place them within a single category of internalizing disorder (ID) whose core symptoms—anxious emotional states, depressed mood, somatization, and social withdrawal—are associated with overcontrolled behaviors and subjective distress. According to the tripartite model of comorbidity, the strong cooccurrence of anxiety and depression symptoms constitute a shared internalizing syndrome, *negative affectivity*, which is linked to other internalizing symptoms such as low positive affect and anxious arousal [4-6]. In this vein, developmental theories describe an internalizing pathway to SUD whereby childhood behavioral challenges marked by negative affectivity and social inhibition predispose adolescents to substance use (SU) problems [7,8].

Longitudinal outcome data suggest that adolescents with cooccurring SUD+ID are more difficult to treat effectively than those who do not have IDs. One systematic review [9] found that adolescents with comorbid SUD and depression showed higher attrition from treatment, and generally worse outcomes for both SU and depression, than those with a singular disorder. Another study [10] reported that baseline levels of elevated ID symptoms or diagnosis of an anxiety or depression disorder broadly predicted worse SU outcomes. Studies have also shown that maintenance of SUD treatment gains [11,12] and posttreatment recovery to adaptive levels of functioning [13] are harder to achieve for adolescents with IDs and related negative affect. It also appears that ID-related symptoms impact posttreatment SU behavior in multiple ways, foremost being that negative affect can induce adolescents to reinitiate use [14] or interfere with their motivation and capacity to use relapse prevention and health promotion skills learned during treatment [15].

### Usual Care for Adolescent SUD: Sizable Gaps in Evidence-Based Treatment Planning for ID

A recent Substance Abuse and Mental Health Services Administration resource guide on adolescents with serious emotional disorders and cooccurring SU [16] asserts that integrated planning for adolescent SUD+ID is routinely inaccessible due to several factors, including broad service fragmentation and limited cross-training for both SU and mental health clinicians; moreover, few clinicians have specialized training in both disorders. Clinicians with specialty licenses to treat SUD are typically required to obtain only generic training in cooccurring disorders; they do not receive systematic training in IDs or certified training in evidence-based practices for ID [17]. Surveys of the adolescent SUD specialty workforce confirm this ID services gap [18,19]. One study [20] found that while only 10% of SUD clinicians reported using specific protocols to treat SUD+ID, 97% indicated they would use such a protocol, and moreover, they could dedicate 8 workday hours to train in it.

### Integrated Treatment Models for Comorbid SUD+ID: Major Barriers to Implementation

One strategy to promote treatment planning and training for SUD+ID is to disseminate integrated treatment models that contain 2 components: one targeting SUD and the other targeting ID. However, because integrated models of this type require multidomain symptom targeting and intervention coordination, they are difficult to implement and evaluate [21]. To date, only a few integrated models for adolescent SUD+ID have been tested in randomized trials. Esposito-Smythers et al [22] demonstrated the effectiveness of a protocol that included cognitive behavioral therapy (CBT) for SUD, individual CBT for depression and suicidality, and family skills training. Rohde et al [23] varied treatment delivery sequence for adolescents with SUD and depression. This led to the finding that compared to a CBT-first model and a simultaneous family-and-CBT model, family therapy for SUD followed by CBT for depression was the most effective for combined SU and depression outcomes. Goldston et al [24] found that a protocol grounded in CBT and relapse prevention for SUD, depression, and suicidal behavior outperformed usual care. Two other manualized models [25-27] integrated family and individual CBT interventions for SUD and ID, including posttraumatic stress disorder; a handful of studies have examined brief interventions targeting both SU and ID in adolescents [28].

Unfortunately, none of the integrated models targeting adolescent SUD+ID has been scaled to widespread use. A first category of scalability barriers pertains to *characteristics of the behavioral health system*. Clinicians with specialty SUD licenses typically have little training or clinical support in addressing IDs. Factors related to clinician training, site licensing, and health care regulation contribute to this deficiency [16,29]. As a result, adolescent SUD clinicians are generally not familiar with evidence-based ID interventions and are not clinically prepared to deliver them [20,30]. This deficiency reflects a broader and longstanding service fragmentation problem in the United States: mental health and SU treatment systems are largely siloed from one another, failing to adequately address the needs of adolescents with cooccurring problems [31,32]. A second category of scalability barriers pertains to *characteristics of integrated models*. Previously described integrated SUD+ID models share basic structural features with the larger phylum of manualized behavioral models. Manualized models typically feature structured intervention sequencing and wholesale implementation of treatment components. In contrast, community clinicians favor piecemeal implementation and selective treatment planning, including the flexible use of techniques as adjunctive interventions for cases with complex diagnoses [33,34]. Manualized models also contain extensive quality procedures to facilitate therapist training, fidelity, and ongoing certification. Such procedures are difficult to sustain due to vicissitudes in regulatory practices, decrease in agency stamina to honor quality methods for extended periods, and demoralization when external experts manage quality control [35].

### Promising Solution to Overcoming Implementation Barriers: Core Elements Strategy

Formidable implementation barriers negatively impact the proficiency of disseminating and training the SUD workforce to use integrated models for SUD+ID. Similarly, it will not be enough to more efficiently disseminate and train SUD clinicians in manualized models for adolescent depression, anxiety, or both, all the same barriers to widespread scalability loom. An alternative strategy to advance scalability of evidence-based treatments is to focus on core elements that are common across models for similar populations. The core elements strategy defines reduced sets of specific techniques that are common, active ingredients in multiple treatments for a given disorder [34,36]. This strategy offers implementation advantages in 2 key domains: *modularity and sustainability*. Regarding *modularity*: core elements, delivered as modular units or as a sequence of discrete interventions, can be more easily learned and retained by trainees and can be flexibly selected and coordinated to address diverse, comorbid, and emerging clinical problems [37,38]. Regarding *sustainability*, core elements promote clinician judgment, coupled with broad guidelines, to flexibly select interventions to fit client profiles [39]; provide strategies to select interventions for comorbid clients for whom no dedicated manuals exist [37,40]; and avail a few intervention modules to apply across numerous clients [34,36]. Core elements have proven feasible to scale in routine settings [37,41,42]; moreover, core elements can perform on equal footing with manualized treatments across an array of adolescent outcomes [41,43,44].

### Core Elements for Targeting IDs Among Adolescents With SUD+ID: 3 Foundations

#### Overview

**Figure 1:**
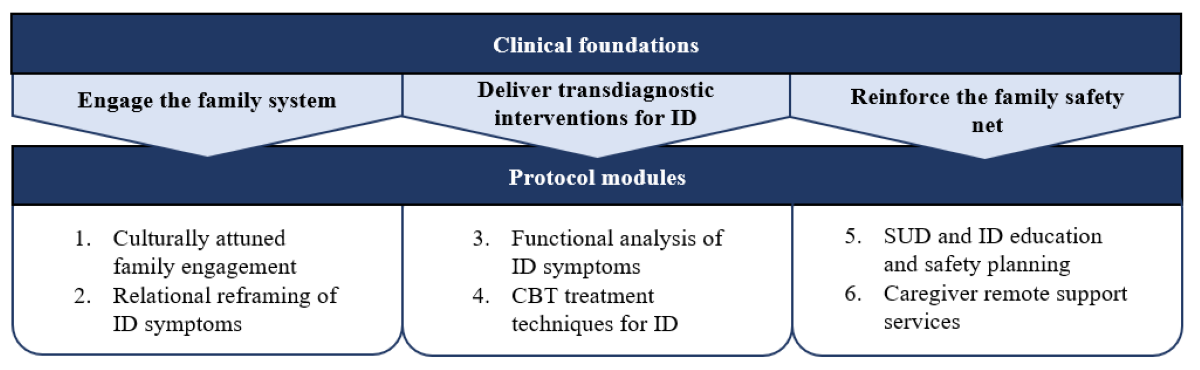
Protocol foundations for this study. CBT: cognitive behavioral therapy; ID: internalizing disorder; SUD: substance use disorder.

In line with the core elements strategy, this study is developing a core elements protocol designed to treat ID symptoms among adolescents with SUD in usual care. It combines several existing treatment techniques, most of which have empirical support for addressing treatment targets related to adolescent ID, into a single modular protocol: Family Support Protocol for Adolescent Internalizing Disorders (Fam-AID). As depicted in Figure 1, intervention selection is guided by 3 broad evidence-based principles for treating adolescent SUD+ID: Engage the Family System, Deliver Transdiagnostic Interventions for ID, Reinforce the Family Safety Net.

#### Engage the Family System

To enhance adolescent readiness to participate in behavioral treatment of any type, it is highly advantageous to engage parents and other family members actively [45]. Research-based family engagement interventions for an array of adolescent services include emphasizing the role of family involvement in services, anticipating how family resources and dynamics could impact participation, building alliances with multiple family members, and managing family interactions during initial clinical encounters [46]. Specific to adolescents, evidence-based interventions for engaging families include systemic family engagement [47-49], which focuses on recognizing incompatible agendas of family members and how this reduces the likelihood of any member attending, identifying who can act as a reliable family messenger and who has power to influence other members to attend, and providing rationale for treatment that accounts for concerns of key members. An engagement strategy unique to family-based treatment is relational reframing [50,51], which involves efforts to transform symptom- or person-focused perceptions of clinical problems into an understanding of those problems as having important, fundamentally relational aspects [52-55].

#### Deliver Transdiagnostic Interventions for ID

Adolescents with elevated ID levels often have underlying emotional vulnerabilities in 3 areas: negative emotional reactivity, in the form of more frequent experiences of intense negative emotions and less flexible reactions to these; affect intolerance, such that emotional experiences are perceived as difficult to endure; and emotional avoidance, wherein adolescent respond to affect intolerance with efforts to suppress, avoid, or otherwise control uncomfortable feelings [56,57]. Therefore, interventions for adolescent ID need to target high levels of negative affect, distress intolerance, and affect avoidance. An efficient approach for treating these overlapping vulnerabilities is to use transdiagnostic interventions, which treat similar behavior problems (eg, anxiety and depression) via interventions targeting higher-order precipitating processes (eg, emotion dysregulation) [58,59]. Transdiagnostic interventions have proven efficacious for adolescents with IDs in multiple baseline, open, and randomized trials [60-63], producing improvements in depression, anxiety, and emotion dysregulation symptoms [60,61,64]. Ideally, transdiagnostic interventions for ID symptoms among adolescents with comorbid SUD+ID should be guided by functional assessment of the adolescent’s specific ID-related impairments [21], which facilitates treatment customization by narrowing the field of available techniques to those specifically indicated for the client [65-67]. Of the several transdiagnostic techniques available for adolescent ID, 3 appear most salient for addressing factors that also predispose SU among adolescents: *emotion acceptance*, which targets emotional regulation and distress tolerance and also enhances inhibitory learning during exposure [60,61,64,68]; *emotional exposure*, a primary driver of positive effects for adolescent anxiety [69]; and *behavioral activation*, a primary driver of positive effects for adolescent depression [70].

#### Reinforce the Family Safety Net

An essential component of comprehensive treatment planning for adolescents with SUD+ID is accounting for potential self-harm, suicidal ideation, and suicidal behavior, which often occur among adolescents with clinically elevated ID levels, especially when there is cooccurring SUD [71,72]. Adolescents and their families can be co-educated about potential self-injurious behaviors and invited to cocreate safety plans [73]. Although single-session adolescent-only safety contracts are not routinely effective, more intensive safety planning involving families can reduce self-harming behavior in adolescents [74]. Moreover, family members can be sources of recovery capital to help adolescents sustain reductions in SUD and ID symptoms.

### Specific Aims of the Study

In year 1, this study will develop a Fam-AID implementation toolkit during a three-part *pilot stage* at 1 pilot site by (1) soliciting provider input on Fam-AID components; (2) creating video-based training and fidelity procedures, leveraging the research team’s existing training and consultation resources in both core family-based interventions [75,76] and adolescent-focused transdiagnostic techniques [77] for adolescent behavior problems; and (3) piloting the toolkit with 4 to 6 clients. In years 2 to 3, this study will conduct an *interrupted time series* evaluation for 60 adolescent SUD+ID cases across 2 sites serving diverse clients: 30 cases will receive treatment as usual (TAU), and then following clinician training, 30 new cases will receive TAU enhanced by Fam-AID.

This protocol development study has 2 specific aims:

- Aim 1 (protocol feasibility): in the Fam-AID condition only, the study will evaluate (1) protocol acceptability via client and therapist qualitative interviews and (2) fidelity benchmarks via therapist- and observer-report fidelity measures of protocol coverage (Is each module delivered in at least 1 session?) and protocol dose (Is the average module extensiveness score greater than the mean of the fidelity measure?).
- Aim 2 (protocol outcomes): in comparing the TAU only condition versus the TAU+Fam-AID condition, the study will examine the protocol’s impacts on (1) family member session attendance using clinic logs and (2) adolescent ID-related symptoms (emotion regulation, depression, and anxiety), SU, and family communication about SUD+ID at 3-month and 6-month follow-up. Overall, these pilot-scale protocol viability and impact findings, if promising, will set the stage for a randomized trial to examine the effects of Fam-AID on immediate and ultimate outcomes in a larger community sample.

## Methods

### Pilot Stage: 3-Phase Clinical Protocol Compilation Procedures

At *project initiation* (months 1 and 2), the study team will review three clinical protocols and companion training resources that were collectively produced by them: (1) archive of empirically distilled core family therapy treatment techniques for adolescent SUD [51], (2) adjunctive family-based protocols for targeting cooccurring attention-deficit/hyperactivity disorder among adolescents with primary SUD [78,79], and (3) the Unified Protocol for Adolescents for transdiagnostic CBT interventions to treat adolescent ID [60,77,80]. The team will converge these resources to create prototype components for the 5 Fam-AID modules. They will then launch the pilot stage, a 3-phase stakeholder-informed process to develop Fam-AID prototype components into a beta protocol. Pilot phase work will follow procedures of rapid-cycle prototyping that use mini-pilots to test early versions of protocols, combined with ongoing feedback from content experts and other stakeholders, to learn how to best design new strategies [81,82].

During *pilot phase 1* (study months 3 to 5), the study team will conduct in-depth interviews with clinic staff and families (adolescent and caregivers) at 1 pilot site, with 3 primary goals. First, they will interview both staff and families about existing barriers and potential facilitators to engaging caregivers in site services. Though evidence-based interventions exist for engaging families in adolescent services, and these will be systematically incorporated in Fam-AID module 1, such interventions are rarely used in routine care. To help prepare module 1 for this challenge, the team will apply their expertise in this area [83] to identify and investigate engagement barriers (logistic, attitudinal, and institutional) and generate pragmatic strategies to solve or ameliorate barriers and enhance both (a) family motivation and intention to participate and (b) site policies to facilitate family involvement. This information will feed forward into pilot phase 2 and phase 3 activities and ultimately into the design of the new protocol. Second, the team will confer with site administrators and staff to obtain feedback on proposed clinical and fidelity strategies for engaging the family system through culturally attuned engagement strategies and relational reframing of the adolescent’s ID symptoms, delivering transdiagnostic interventions for adolescent ID using in-session functional analysis of ID symptoms followed by the selection of CBT techniques for ID, and reinforcing the family safety net via family-based psychoeducation about SUD+ID symptoms and long-term safety planning interventions related to adolescent self-harm. Third, they will interview 3 to 4 adolescents and families to canvass their knowledge, attitudes, social influences, and personal experiences related to SUD and ID services, with emphasis on perceived barriers and facilitators to integrated treatment planning for ID symptoms. The milestones for the completion of pilot phase 1 will be collaborative team-clinic consensus on initial drafts of enhanced family engagement strategies, overall protocol content, and training and implementation materials for the projected 5 Fam-AID modules.

During *pilot phase 2* (months 6 to 11), guided by phase 1 results, the team will finalize modifications to Fam-AID component prototypes. The team will subject prototype materials to cognitive interviewing [84] with staff and clients at the pilot site, during which respondents will be asked to think aloud when answering questions about components. Cognitive interviewing is used to refine procedures by assessing respondent comprehension, what is meant by a particular response, and whether additional content is needed to complete a process. This phase will also focus on usability testing of protocol and implementation materials to ensure they are user friendly and efficient in delivering specified interventions [85,86]. The milestones for pilot phase 2 completion will be rapid-cycle consensus on modifications to Fam-AID prototype components, resulting in a beta protocol that accounts for barriers and facilitators to delivery in routine care, which will then be vetted in pilot phase 3.

During *pilot phase 3* (months 12 to 14), the team will pilot the beta Fam-AID version by delivering components to 2 to 3 clients, successively adjusting component prototypes across clients and sessions, to tweak module design and troubleshoot clinical and technological features. They will then finalize component prototypes through culminating interviews with staff and clients to vet feasibility, utility, and resource requirements; this will help with empirical optimization of delivery parameters (dose, frequency, and sequencing benchmarks). The milestone for pilot phase 3 completion is rapid-cycle finalization of components constituting the Fam-AID modules.

### Interrupted Time Series: Study Design

**Figure 2:**
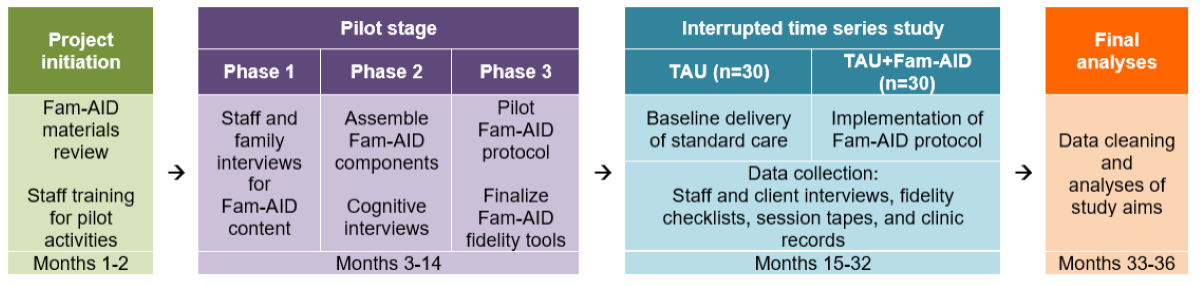
Design and timeline of study activities for this study. TAU: treatment as usual.

Following the completion of the pilot stage, the team will conduct an interrupted time series study featuring quasi-experimental pilot effectiveness research methods to evaluate the Fam-AID protocol and implementation toolkit [87]. Figure 2 depicts the basic design and timeline of the interrupted times series study. The quasi-experimental interrupted time series with nonequivalent groups, which tracks target data both before and after introducing a programmatic change to a group whose membership may vary over time, is appropriate for testing viability and impacts of new interventions delivered in field settings [88-90]. This design is functionally similar to a posttest-only design with a nonequivalent historical control group. Two partner clinic sites will be involved to reduce confounds from site-specific implementation effects and increase diversity in clients and therapists. Aim 1 methods will feature procedures designed for stage 1 treatment development [91,92] to establish proof-of-concept (acceptability and fidelity benchmarks) for the protocol and toolkit. Aim 2 methods will examine Fam-AID’s impact on family member attendance in treatment and client outcomes (adolescent ID symptoms, adolescent SU, and parent-adolescent communication about SUD+ID).

### Study Sites and Participants

Both study sites are licensed to deliver SUD services to adolescents. Neither site currently supports an evidence-based treatment protocol that specifically targets IDs. Participant inclusion and exclusion criteria are shown in Textbox 1.

**Participant inclusion and exclusion criteria.**

**Inclusion criteria**

- The adolescent is aged 13 to 18 years.
- The adolescent lives with a primary caregiver who can attend treatment sessions.
- The adolescent endorses ≥1 *Diagnostic and Statistical Manual of Mental Disorders, Fifth Edition, Text Revision* [93] symptom for substance use disorder and meets the American Society of Addiction Medicine criteria for outpatient substance use treatment.
- The adolescent meets the *Diagnostic and Statistical Manual of Mental Disorders, Fifth Edition, Text Revision* criteria, or has elevated symptoms and impairment, for an ID, with the following expected to be most common: current or recurrent major depressive episode, pervasive depressive disorder, social anxiety disorder, generalized anxiety disorder, panic disorder, and posttraumatic stress disorder.
- The adolescent completes intake and is enrolled as an active case at the study site.

**Exclusion criteria**

- The adolescent has an illness requiring hospitalization.
- The adolescent has current psychotic symptoms.
- The adolescent has severe substance use problems that require immediate relief (detox or residential placement).
- The adolescent has pervasive developmental disorder.

To match real-world care, no eligibility criteria or other study directives will pertain to medication status. Assessment data collected during previous studies in the same regions [79,94] provide the following adolescent demographic projections: average age approximately 16 years; male participants, 70% (42/60) and female participants, 30% (18/60); Black, 15% (9/60); Latinx, 40% (24/60); White non-Latinx, 40% (24/60); and other, 5% (3/60).

### Study Condition: TAU Only

Previous research on TAU intervention delivery at both study sites using therapist self-report [95] and observational data from session recordings [96] indicates that site therapists routinely deliver relatively low-dose quantities of evidence-based SUD treatment techniques, favoring motivational interviewing and addiction counseling techniques, with even less delivery of CBT or family therapy techniques for SUD. Sites do not have existing protocols for treating IDs as either main referral problems or cooccurring conditions.

### Study Condition: TAU+Fam-AID

#### Overview

Fam-AID will contain 5 modular components to be delivered in any order and to the extent indicated based on case status [37,43,97]. Modules can be individually or collectively completed in 1 session, staggered across sessions, and interspersed with other interventions. The time required to complete each module will vary based on client profile, practice habits of the provider, and case progress. Fam-AID will have standardized options for versatile delivery with individual adolescents alone whenever family members cannot be engaged in services; modules 3 and 4 can proceed intact, and module 5 can be adjusted for adolescent-only delivery. The study will be halted if it appears that Fam-AID delivery induces clinical harm. The following is a description of the projected module content, considering that the ultimate set of modules and their content will be determined over the course of the study:

#### Module 1: Culturally Attuned Family Engagement

To promote family engagement, clinicians collaborate with the family members by instilling hope and involving them in goals related to ID problems. They encourage the family members to attend sessions throughout care, present treatment as an opportunity to talk about new issues or old issues in new ways, connect with the family members by showing respect and validation, and use relevant self-disclosure to establish connections [98-100]. Family engagement is guided by the principles of cultural attunement [101], which include accentuating values and strengths minimized in dominant culture, remaining accountable for how interventions disrupt or reinforce existing inequities, and encouraging the family members to make conscious choices about how they frame their identities and respond to injustice.

#### Module 2: Relational Reframing of ID Symptoms

Clinicians use relational reframing techniques to shift the focus of treatment from exclusively fixing adolescents and their symptoms to improving the quality of adolescent-family relations. They assert that treatment should be grounded in acknowledging, understanding, and repairing relationship problems as a means of addressing adolescent ID problems and bolstering individual and family recovery paths. Basic approaches to delivering a relational reframe for adolescent IDs include identifying sequences of behaviors or emotions involving families that precede or directly cause identified problems; focusing on the impact that ID-related problems have on the thoughts, feelings, and behaviors of both adolescent and family; and supporting relational repair or improvement [52-55].

#### Module 3: Functional Analysis of ID Symptoms

Clinicians use functional analysis to uncover information about reinforcers of symptoms to render interventions that are personally relevant as well as help adolescents and families understand behavioral chains of antecedents and consequences that maintain problems. Functional analysis steps for adolescent ID include [102-104] articulating scenarios in which ID-related problems occur; examining circumstances that precede problems, ascertaining both internal and external antecedents; identifying short-term positive outcomes and long-term negative outcomes; and engaging clients in comparing positive versus negative consequences. Clinicians are careful to validate feelings that emerge, reflect appreciation for client sharing, and maintain a stance of curiosity about the perceived value of behaviors and an empathic position in exploring healthier alternatives.

#### Module 4: CBT Treatment Techniques for ID

Clinicians use data from the functional analysis of ID symptoms to choose from a selection of 3 transdiagnostic CBT techniques that have evidence for treating both depression and anxiety in teens [105,106]. In *emotional acceptance*, adolescents develop skills for noticing and accepting their emotions, including unpleasant and strong emotions [60,61,64,68], which help reduce anxiety sensitivity. They learn skills of present-moment and nonjudgmental awareness, which can increase affect tolerance [107]. In both *emotional exposure* and *behavioral activation*, adolescents are helped to approach situations, thoughts, and triggers that elicit strong emotions that have been previously avoided [60,61,64,102,104,108,109]. They try out various “opposite” actions to their typical emotional responses or behaviors (eg, volunteering to speak in class) to increase distress tolerance. Adolescents are encouraged to use new skills to fully engage in situations eliciting strong emotions and maintain clarity about the purpose of the opposite actions they select. All 3 techniques operate primarily on an approach versus avoidance clinical mechanism.

#### Module 5: SUD+ID Education and Safety Planning

Clinicians use family decision coaching [79,110,111] to converse with family members about three overlapping pathways of SUD and ID cooccurrence [10,112-114]: (1) self-medication: ID precedes the onset of SUD, and substances are valued for psychoactive properties that alleviate ID symptoms; (2) SU exposure: SUD precedes the onset of ID and creates developmental lag in the emergence of self-regulatory functions that precipitate ID and, if severe, impair neurocognitive functioning; (3) shared etiology: SUD and ID are mutually influencing axes of dysregulation that compromise healthy development. Family SUD+ID education transitions to family-based safety planning about both nonsuicidal self-injury and suicide ideation and attempts, guidelines for which focus on establishing physical and psychological safety, identifying strategies that can be used instead of self-harm, and identifying individuals to whom the adolescent can go for help [73,74,115,116].

### Fam-AID Training and Fidelity Monitoring

Site clinicians will be trained for 6 hours. The session will feature video-based training [117,118] in Fam-AID modules and techniques. Thereafter, consultation at each site will include bimonthly live consultation with a protocol expert focused on equipoise between protocol fidelity and case tailoring, along with continuous feedback from therapist- and observer-report fidelity data, which is emailed directly to clinicians and used in live consultation to enable rapid adjustment in protocol delivery [119-121]. Two main fidelity benchmarks will be monitored for each case. These benchmarks include clinicians delivering interventions for every module in at least 1 treatment session (ie, coverage) and clinicians performing at or above the mean scale score for intervention extensiveness on the Fam-AID Fidelity Checklist (described in the Study Measures section) for at least 1 technique from each module (ie, dose), with both therapist- and observer-report checklist versions being tracked.

### Study Procedures

All full-time therapists at both study sites who volunteer to participate will be consented. We project 4 therapists at each site (N=8) will participate. As in the authors’ previous work [79], clinic staff will discuss the research opportunity with the families of all the adolescents during routine intake procedures. If consent is given, clinic staff will send the family’s contact information to the study team through secure channels. The study team will contact interested caregivers by phone to complete (1) consent to phone screen, audio record sessions, complete outcome interviews and questionnaires, and grant access to clinic records and (2) an eligibility screener assessing IDs. IDs will be assessed using the Achenbach System of Empirically Based Assessment Child Behavior Checklist [122], a well-validated assessment method for screening for depression and anxiety problems in youth. IDs will also be assessed during the baseline research assessment using the Mini-International Neuropsychiatric Interview for Children and Adolescents [123,124], a structured diagnostic interview administered by lay interviewers; the interview yields Diagnostic and Statistical Manual of Mental Disorders, Fifth Edition, Text Revision categories and has been used in diverse adolescent samples [79,125]. Client data on protocol viability (limited to Fam-AID cases only) and clinical outcomes will then be collected on all study clients (regardless of amount of treatment participation) at baseline, 3-month, and 6-month follow-up using REDCap (Research Electronic Data Capture; Vanderbilt University) cloud software, a widely used and secure Health Insurance Portability and Accountability Act (HIPAA)–compliant, web-based data collection platform.

Clinicians in both conditions will be asked to complete a self-report fidelity measure after every session with a study client. They will also be asked to audio record all sessions, a minimally intrusive procedure that is widely accepted by families and therapists in previous work [94]. Data will be collected using web-based links and uploaded to secure servers. As per the study team’s numerous observational fidelity studies [126-130], 1 session will be randomly selected from the early phase of treatment (sessions 1 to 3) and 2 from the later phase (sessions from session 5) of each client for coding with an observational version of the Fam-AID Fidelity Checklist (described in the Study Measures section). Fidelity observational coders will be trained by the study team using gold standard procedures [126,131-133] and kept naive to the study condition.

### Study Measures

#### Fam-AID Fidelity Checklist

This study will use an adapted version of the Inventory of Therapy Techniques (ITT [95,96]), a postsession therapist-report fidelity tool that requires 1 or 2 minutes to complete. ITT items are designed to measure the extensiveness (ie, thoroughness and frequency) with which a given treatment technique was used on a 3-point Likert-type scale: 0 (not at all), 1 (a little bit or moderately), or 2 (quite a bit or extensively). ITT items are derived from treatment protocols and their associated observational fidelity tools, for example, for family therapy [51] and for CBT [67], after being vetted for relevance by community clinicians in multiple treatment settings [134]. ITT has shown strong construct [50,134] and predictive [79,135] validity in studies with adolescents receiving family therapy and CBT in usual care. The Fam-AID Fidelity Checklist mimics the ITT structure, containing multiple items in each of the 5 Fam-AID modules: culturally attuned family engagement, relational reframing of ID symptoms, functional analysis of ID symptoms, CBT treatment techniques for ID, and SUD+ID education and safety planning. The Fam-AID Fidelity Checklist–Observer Version is an identical measure with instructions for observer coding.

#### Protocol Acceptability

Protocol acceptability interviews will be held with therapists and clients who participate in the Fam-AID protocol. Therapists will be interviewed in agency-based focus groups. Clients will be interviewed at the 6-month follow-up assessment point; adolescents and caregivers will be interviewed separately. Interview content will be adapted from existing treatment feasibility and acceptability interviews (refer to the study by Cho et al [136]) and include the following questions:

- What are the overall impressions of Fam-AID for use in routine practice (therapist; open-ended)?
- How effective and/or difficult it was to use Fam-AID for individual cases compared to usual practice? (therapist, Likert-type rating)
- Was it important for you (your caregiver) to be included in treatment? (client, open-ended)
- How much did emotion coping training help you (your child)? (client, Likert-type rating)

#### Adolescent and Family Outcomes

Clinical record data will be collected on the number of treatment sessions attended by family members, along with the data on adolescent attendance. Difficulties in Emotion Regulation Scale [137,138] is a 16-item measure of 6 aspects of emotion regulation: nonacceptance, engagement in goal-directed activities, impulsivity, emotional awareness, access to emotion regulation strategies, and emotional clarity. It uses a 5-point Likert scale and has shown acceptable test-retest reliability and internal consistency in adolescent populations [137-140]. The Revised Children’s Anxiety and Depression Scale-Child and Parent version [141] is a 47-item measure of an adolescent’s anxiety and depressive symptoms. The Revised Children’s Anxiety and Depression Scale-Child version contains 6 *Diagnostic and Statistical Manual of Mental Disorders, Fifth Edition, Text Revision*–linked subscales: separation anxiety, social anxiety, generalized anxiety, obsessive-compulsive disorder, panic disorder, and major depressive disorder. A total anxiety subscale score sums 37 items from the 5 anxiety subscales; the major depressive disorder subscale score sums 10 items. The Revised Children’s Anxiety and Depression Scale-Parent version is identical to the Revised Children’s Anxiety and Depression Scale-Child version, but the wording of the items is from the parents’ perspective. The Revised Children’s Anxiety and Depression Scale-Child and Parent version have exhibited strong and acceptable internal consistency, respectively [142-144]. CRAFFT [145,146] is a widely used and well-validated adolescent-report tool that measures patient use of alcohol, cannabis, nicotine, illegal drugs, prescription medication, or anything else to get high in the past year and the past 3 months. It also asks about riding in a car driven by someone (including self) who was intoxicated. If SU is reported, the tool asks 5 additional questions: use to relax, use while alone, forget things you did while intoxicated, family or friends tell you to reduce use, and gotten into trouble while using. Parent-Teen SUD+ID Communication Frequency [147] is a 6-item adolescent and caregiver report of how often parents and teens talk to one another about key SUD-ID issues (eg, health risks of SU and ID and discipline regarding SU); response are scored on a 5-point scale, ranging from *never* to *very often* (refer to the studies by Sherman and Ehrenreich [77], Hogue et al [78], and Hogue et al [79]). Parent-Teen SUD+ID Communication Quality [148] is a 6-item adolescent and caregiver report on the quality of communication between parent and adolescent about SUD+ID issues (eg, my caregiver/child and I are interested in each other’s opinions about SUD+ID, and if my caregiver/child and I talk about SUD+ID, I feel understood); responses are scored on a 5-point scale, ranging from *not at all* to *very much* (refer to the studies by Koning et al [147] and Carver et al [149]).

### Plan of Analyses

*Aim 1 acceptability* analyses (Fam-AID condition only) will explore protocol acceptability and perceived effectiveness through formative qualitative interviews with staff and clients. Interviews will be recorded, transcribed, and then analyzed inductively [150] for data relevant to feasibility and acceptability. Two research staff members will separately code interviews for emerging concepts using thematic content analysis [151], an approach that applies inductive coding to identify themes within the data. Initial analysis will identify concepts through open coding [152]; inter-rater reliability checks will then be used to compare themes generated and resolve conflicts, and coding will continue to the point of saturation, that is, when no new concepts emerge.

*Aim 1 fidelity benchmarks* analyses (Fam-AID condition only) will examine both types of previously described fidelity metrics. Regarding coverage, descriptive statistics will capture frequencies related to (1) proportion of clients who received at least 1 intervention from each of the 5 modules and (2) interventions from which modules were most or least favored across clients. Chi-square analysis will be used to compare study conditions on the proportion of clients who received adequate coverage and conduct one-way analysis of variance to assess mean differences across modules in the Fam-AID condition to evaluate module preferences among therapists. Regarding dose, statistical equivalence testing [153] will be used to examine whether clinicians’ average scores for intervention extensiveness on both observer- and therapist-report versions of the Fam-AID Fidelity Checklist are equivalent to a designated benchmark, which will be set (granting appropriate latitude for a pilot trial) for each module as mean 2.5 (SD 0.50). A CI approach will be adopted in which an equivalence interval is defined as the benchmark +10% and –10%. Next, a CI is defined by the following formula: CI_90%_=M_R_–M_T_+/–z_α_(S_MR−MT_). In this equation MR represents the benchmark module mean, MT the average module score for study therapists, zα the critical one-tailed value from the z distribution for the chosen value of α, and SMR−MT the pooled SE. If the calculated CI falls within the equivalence interval, equivalence can be concluded. In addition, therapist variability in Fam-AID fidelity will be examined via statistical process control analyses (refer to the study by Hogue and Dauber [125]) in which individual therapist fidelity scores are plotted on a control chart to check for meaningful variation compared to control limits represented by the designated fidelity benchmark.

*Aim 2a family attendance* will involve between-condition comparison of treatment sessions attended by family members other than the adolescent. Linear mixed effects (LME) modeling will be used to examine the effect of condition on attendance counts. The model will include fixed effects for independent variables (ie, condition) and random effects to account for nesting at the therapist level, thereby providing unbiased parameter estimates [154,155]. Nesting at the client level will not need to be controlled in these analyses, as client summed totals will serve as a dependent measure: tallies of sessions attended by family members will be summed across the follow-up period. The LME models package [156] will be used; it provides full information maximum likelihood estimation and restricted maximum likelihood estimation, both of which produce unbiased parameter estimates based on the assumption that data are missing at random and outperform other missing data approaches even when missing at random is not met [157]. Effect sizes will be indexed by η_2_ [158]. However, the nonlinear mixed effects model will be used if the assumption of continuous data is not tenable (eg, when attendance counts are not normally distributed).

*Aim 2b adolescent and family outcomes* analyses will involve similar LME models previously described. To test for change over time, each model will include fixed effects for time (months since baseline, coded as 0, 3, and 6) and random effects for (1) nesting of clients within therapists and (2) repeated measures within person across follow-up. Hypotheses will be tested by adding condition as a fixed effect and examining the condition-by-time interaction on each outcome in separate models. Acknowledging that outcomes may not demonstrate sharp trajectory change during the relatively brief 6-month follow-up window, the intercept will be set at the terminal assessment point by recoding time points (–6, –3, and 0); in doing so, the condition effect for the intercept will reflect a between-group difference at 6-month follow-up. LME modeling is recommended as an alternative to traditional modeling techniques for longitudinal designs with small samples [159].

### Study Power

Aim 2 analyses will compare study conditions based on family attendance and client outcomes over time, with client (N=60) as the unit of analysis. Assuming α=.05, a total sample size of N=60 (with 30 clients per condition), 3 measurement occasions with a moderate intercorrelation (*r*=0.50), and a moderate effect size of Cohen *d*=0.60, the analysis yields a power of 0.80. A smaller effect size of Cohen *d*=0.50 yielded a power of 0.65, assuming α=.05. These estimates are in line with the impacts seen in the studies on adolescents with ID but not SUD [60,62] and in the studies on adolescents with SU problems treated in usual care [79,94].

### Ethical Considerations

Ethics approval for this trial is pending from Solutions Institutional Review Board. Adolescents and caregivers will independently provide informed assent or consent before the initiation of the study activities (Multimedia Appendix 1). All study activities will be subjected to monitoring by the Data Safety and Monitoring Board of the same institution (Multimedia Appendix 2). Any modifications to the protocol that might impact the conduct of the study or its specified objectives and procedures will require a formal amendment to the protocol and approval by the Institutional Review Board and the Data and Safety and Monitoring Board before implementation. As reimbursement for interview completion, each participant receives a gift voucher (of their choice) worth US $110 via Tango Card: US $30 each for participation at baseline and US $40 each for participation during 3-month and 6-month follow-up. All assessment data will be automatically downloaded from the web-based data collection portal into a secure database maintained in a secure firewall- and password-protected location on a data network. All network data are backed up daily. Only project staff will have direct access to the data. All standard procedures for data management and security will be followed. For planned statistical analyses, database managers will extract data from the REDCap repository. All participant identifiers will be removed before the data are provided to statisticians for analysis.

## Results

This study is being conducted over a 3-year period. Participant recruitment and data collection will begin in April 2025. We expect results from this study to be published in 2027.

## Discussion

### Overview

This pilot treatment development study will test feasibility (acceptability and fidelity) and short-term client outcomes (adolescent ID symptoms, adolescent SU, and family communication about SUD+ID) of a modular protocol for addressing cooccurring IDs among adolescents with SU in routine care. Fam-AID will contain 5 modules that can be delivered in any sequence to meet client needs: *family engagement* in treatment planning and services; *relational reframing* of family constraints, resiliencies, and social capital connected to the adolescent’s ID symptoms; *functional analysis* of ID symptoms and related behaviors; *cognitive behavioral therapy* to address ID symptoms and functional needs; and *family psychoeducation and safety planning* focused on education about comorbid SUD+ID and prevention of adolescent self-harm. If shown feasible and effective, Fam-AID will offer a set of pragmatic interventions to SUD clinicians for treating cooccurring IDs in adolescent clients.

Importantly, Fam-AID is not intended to contain components that directly target SUD outcomes. This means that it is designed as an adjunctive intervention to complement and enhance the current SUD interventions, which are already being practiced by a given clinician or agency. The value of adjunctive protocols, such as Fam-AID, is to augment the overall effectiveness of care by addressing prominent cooccurring problems that, if untreated, derail treatment goals for a primary problem. The adjunctive nature of Fam-AID is a key asset for scalability; clinicians are not asked to exchange or markedly alter their base practices for SUD.

Fam-AID contains 5 modules, each consisting of core treatment techniques that have a strong evidence base. In keeping with the principles of modular intervention design [36,160,161], clinicians are invited to select and implement any Fam-AID module, in any order, based on client needs, with a default of delivering them sequentially. The 5 Fam-AID modules are intended to galvanize and reinforce the family support system and equip the adolescent to cope with and ameliorate ID problems. Notably, module 4 draws its menu of core CBT techniques to treat ID-related problems (emotional acceptance, emotional exposure, and behavioral activation) from an existing evidence-based transdiagnostic protocol, which treats the underlying negative affect and emotional dysregulation [77] associated with adolescent IDs. In these ways, Fam-AID is distinct from other adolescent SUD+ID options due to how it combines a core elements strategy, family-focused interventions, and individual CBT techniques, which might enhance the often modest long-term impact of interventions for ID symptoms in usual care [162].

Fam-AID contains 3 additional innovations intended to boost treatment effectiveness for this group identified as highly vulnerable and enhance the feasibility of implementing protocol modules in routine SUD care. First, in line with the core elements strategy, it is intended for use by invested clinicians of any clinical orientation and training background. To be sure, modules 1 and 2 contain techniques drawn from the family therapy approach, whereas modules 3 and 4 techniques derive from CBT. Nevertheless, Fam-AID modules are constructed as basic, step-by-step clinical practice guides that can be adopted and deployed by even entry-level clinicians. Second, it uses evidence-based family engagement techniques to systematically integrate caregivers in the treatment process. Families are not typically centralized in SUD services for adolescents [163] despite the compelling empirical rationale that family-based treatment is a first-line option for adolescent SUD [164], is well established for adolescent depression [165], and is a useful adjunct for adolescent anxiety [166]. Third, it contains a customization feature in which selected CBT techniques are integrated into treatment planning based on a functional ID assessment completed during sessions. In what amounts to a small-scale version of treatment tailoring [66,167], clients and therapists can collaboratively choose which of the 3 CBT techniques (emotional acceptance, emotional exposure, or behavioral activation) is best suited to address the adolescent’s ID symptoms.

### Study Design Limitations and Strengths

Due to the limited number of sites, clinicians, and clients in this study, results will not be generalizable to the broad network of adolescent SUD treatment clinics or SUD clinical workforce. Randomized designs, which provide control against unknown study confounds and allow for reasonable assumptions of causality related to manipulated variables even in small samples, are generally preferable to quasi-experimental interrupted time series designs. However, we decided against using a randomized design for many reasons. It would result in half as many therapists being trained in Fam-AID, which in a small-scale study exacerbates between-therapist variability in protocol fidelity that could dilute proposed effects and reduces therapist generalizability [87,168]. In addition, it introduces the potential for between-condition bleed from training procedures that could nullify protocol effects. In addition, to the degree that usual practices in the study sites (representing the TAU condition) already feature some type of ID-targeted interventions, the interrupted time series design is credibly stringent. This design was also vastly preferred by our partner clinics because it allows all staff to receive Fam-AID training. Importantly, this design can yield hypothesized viability and impact effects even if routine treatment services for SUD are basically inert at either or both study sites.

### Reproducibility and Dissemination Plans

The Fam-AID protocol will be developed to maximize its reproducibility and scalability by featuring a clinically flexible, modular design strengthened by core element techniques. If the study aims are met, the foundation will be laid to test Fam-AID at R01-level scale. Study information and findings will be disseminated through publication of journal articles, presentations at relevant conferences, and reports to relevant stakeholders. If the Fam-AID protocol ultimately demonstrates feasibility and effectiveness, we will use existing state- and national-level clinician training and licensing platforms to ensure the protocol and its companion fidelity tools and procedures are made widely available to agencies and practitioners who serve youth with SUDs.

## Appendix

Multimedia Appendix 1 Sample consent form for this study.

Multimedia Appendix 2 Study committees and teams for this study.

Multimedia Appendix 3 SPIRIT (Standard Protocol Items: Recommendations for Intervention Trials) checklist for this study.

Multimedia Appendix 4 Peer-review report from Interventions to Prevent and Treat Addictions Study Section (IPTA) - Risk, Prevention and Health Behavior Integrated Review Group - Center for Scientific Review (National Institutes of Health, USA).

## Abbreviations

CBT: cognitive behavioral therapy
Fam-AID: Family Support Protocol for Adolescent Internalizing Disorders
HIPAA: Health Insurance Portability and Accountability Act
ID: internalizing disorder
ITT: Inventory of Therapy Techniques
LME: linear mixed effects
REDCap: Research Electronic Data Capture
SU: substance use
SUD: substance use disorder
TAU: treatment as usual

## Data Availability

The data sets generated and analyzed during this study will be archived is the National Institute on Drug Abuse Data Archive.
