# Supplementary material for: Family Support Protocol for Adolescent Internalizing Disorders: Protocol for a Pre-Post Quantitative Treatment Development Study
Source: JMIR Res Protoc. 2024 Sep 16;13:e64332. doi: 10.2196/64332 (PMC11443177; doi:10.2196/64332)
Supplement: Multimedia Appendix 1 [file resprot_v13i1e64332_app1.docx]

**Adolescent Treatment Study: Caregiver Consent**

We are asking you to join a research study. If you agree to join, you will need to sign this Informed Consent form. Informed Consent is a written agreement that shows you are willing to join this study.

This form will explain:

• the purpose of the study

• what you will be asked to do

• the risks and benefits

It will also explain that you do not need to be in this study to continue getting treatment for your child and family at this clinic. By signing this form, you do not give up any legal rights. You and your child will still be able to receive the care to which you are entitled.

**Why have I been asked to be a part of this study?**

The research team is hoping to understand how to improve treatment for adolescents who are experiencing substance use problems and emotional problems like anxiety or depression. You and your child are being asked to join this study because your child is between the ages of 13 and 18 years old, is enrolled in treatment at this clinic, and during clinic intake reported that they experience substance use and emotional problems.

**What is involved in this study?**

If you agree to join the study, you will be asked to complete a digital assessment session now and again 3 and 6 months from now; you will also be asked to complete a live interview 6 months from now. In addition, staff at this clinic will submit information on your child’s treatment experiences, including (1) audio recordings of treatment sessions with you and your child’s consent, (2) information related to treatment techniques used in sessions, and (3) clinic record data on family demographics and case progress. This information will be used to help the study team learn how to improve treatment services for youth.

**Will there be two different groups in this study?**

There are two different phases of the study: (1) a pre-training phase and (2) a post-training in which therapists will have received additional training in treating emotional problems. Study phases will have no effect on you or your child beyond the training received by your child’s therapist. The study phase you belong to will depend on the timing of your child’s enrollment in services. Youth and families seeking treatment during the first study phase will receive services from a therapist who has not yet had additional training. Those seeking treatment during the second study phase will receive services from therapists who completed the additional training.

**How will the data be used?**

Your information will be shared with the research team only. It will be used only for purposes of the study described in this document. Deidentified data will be added to federally funded data archives to advance scientific research across the field. There is a chance your deidentified data and information may be used for future research studies as well.

**Who is responsible for the data collected in this study?**

If I have any questions about the study, my or my child’s participation in this study, or a research-related injury, I may contact the Principal Investigator:

Aaron Hogue, Ph.D.

Partnership to End Addiction, New York, NY 10117

(212) 841-5278; [ahogue@centeronaddiction.org](mailto:ahogue@centeronaddiction.org)

**Study Specifics:**

**Why is this study being done?**

The purpose of this study is to improve care for youth experiencing substance use and emotional problems. The study will examine special techniques for emotional problems.

This study is funded by National Institutes of Health (NIH) from April 2024 through March 2027.

**How long will I be in this study?**

You will be in this study for 6 months. The length or frequency of your child’s treatment will not be affected by joining this study, but will proceed according to their specific treatment needs.

**How many people will take part in this study?**

About 60 youth and their families will join this study. Some of them may be from your clinic.

**Will my sessions be audio recorded?**

After providing their own consent, therapists who join this study will be encouraged to record all sessions with clients who have also joined. This will help the researchers learn how to train therapists to use special techniques in treatment sessions. Session recordings will also provide an accurate depiction of the clinical techniques used in sessions, allowing researchers to study the effectiveness of the training program.

All audio recordings will be kept confidential and will be stored in a secure folder on the Partnership to End Addiction’s network server, with access restricted to the members of the research team. Only research staff will have access to identifying information. Recordings will be listened to only by research staff and will be kept for at least three years after the study concludes. Your recordings will not be labeled with any identifying information, but instead will be identified by random numbers. You can stop participating in the study at any time, and at any time you can ask that your families’ recordings be destroyed.

**What are the risks of this study?**

There is a small risk of loss of privacy from completing digital and live assessments, and from recording sessions where personal information is discussed. There is also risk of feeling uncomfortable with recording during sessions where personal information is discussed. If at any time you feel uncomfortable, you have the option to take a pause, check-in with the therapist, and/or stop recording. This will not result in any penalty or change to your child’s treatment.

**Are there benefits to taking part in this study?**

You may not receive any direct benefit from joining this study. The information we learn from this study may help others who are receiving mental health and/or substance use treatment.

**Will I receive any payment or other monetary benefits?**

You will be paid $45 for completing the first digital assessment session and $50 for completing each of the remaining two digital sessions. You will also be paid $50 for completing the live interview. The maximum compensation you can earn from this study is $195.

**What other options are there?**

You can choose not to join this study. Doing so will not affect the standing of you or your child at this clinic. If you join the study now and then later choose to leave it, you may request that your data be destroyed. If you do not request that your data be destroyed, the data may be included in the aggregate de-identified data sets and/or used in future analyses.

**What about confidentiality?**

We are required to report information about potential abuse of children, or danger to their lives or the lives of others. Otherwise, all information you provide will be kept private and will be used for research purposes only. Identifying information will be collected solely to track participants throughout the study. No identifying information will be attached to study data; all data will be coded with an ID number. All data will be stored in a secure folder on the Partnership to End Addiction’s network that can only be accessed by the research team.

All research staff with access to study data are required to complete a course on protection of human subjects. They are also trained to follow federal guidelines for maintaining the privacy of participants throughout the study. Study results may be published for scientific purposes, but will not include any identifying information. However, any data from your participation in this study may be reviewed by the National Institutes of Health, by Solutions IRB (the body that oversees our protection of study participants), or by the persons running this study. Anyone who inspects your records will also be required to keep your identity private.

**What is a Certificate of Confidentiality?**

Your privacy is protected by a Certificate of Confidentiality from the National Institutes of Health. With this Certificate, the researchers cannot be forced to disclose information that may

identify you, even by a court subpoena, in federal, state, or local civil, criminal, administrative, legislative, or other proceedings. The researchers will use the Certificate to resist any demands for information that would identify you, except as explained below.

The Certificate cannot be used to resist a demand for information from the National Institutes of Health that will be used for auditing or program evaluation of agency funded projects. A Certificate of Confidentiality does not prevent you or a member of your family from voluntarily releasing information about you or your involvement in this study. If an insurer, employer, or other person obtains your written consent to receive research information, then the researchers may not use the Certificate to withhold that information. The Certificate of Confidentiality will not be used to prevent disclosure to state or local authorities in cases of possible abuse or harm as discussed in the above section.

**What are the costs?**

There are no costs for you to participate in this study.

**What are my rights as a participant?**

Taking part in this study is voluntary. You should consent only after you have been given all

the necessary information. You may choose not to take part in this study. You are also free to

leave at any time. Leaving this study will not result in any penalty or loss of benefits to you

or your child. If you have questions about your rights as a research participant or would like to report a negative event related to this study, you may contact Solutions IRB at (855) 226 – 4472 or by email at [participants@solutionsirb.com](mailto:participants@solutionsirb.com).

**Informed Consent Approval**

I have read this document and I agree to participate in the research study conducted by Aaron

Hogue, Ph.D. If, however, at any time I wish to terminate my participation in this study I have the right to do so without penalty, even after the data have been collected.

I have read and understood this Consent Form, and I agree to participate in this research

study. I give the research team permission to present this work in written and oral form, without further permission from me. I understand that I will be given a digital copy of this form.

Do you agree to participate in this study?

___Yes

___No

Do you agree to the audio recording of sessions for the purposes of this study?

___Yes

___No

_____________________________________ __________________________________

Printed Name Signature

_________________

Date
