# Supplementary material for: Family Support Protocol for Adolescent Internalizing Disorders: Protocol for a Pre-Post Quantitative Treatment Development Study
Source: JMIR Res Protoc. 2024 Sep 16;13:e64332. doi: 10.2196/64332 (PMC11443177; doi:10.2196/64332)
Supplement: Multimedia Appendix 2 [file resprot_v13i1e64332_app2.docx]

Appendix 2

**Study Personnel and DSMB Considerations**

**Principal Investigator (PI)**

Design and conduct of the study

Preparation of protocol and revisions

Publication of study reports

**Steering Committee**

(see title page for members)

Approve final protocol

All study investigators serve as steering committee members

Recruit clinics with the PI

Review study progress, and if necessary, approve changes to the protocol to facilitate study implementation.

**Data Manager Study Coordinator**

Maintenance of study IT system and data entry

Data verification

**Local Champions & Site Research Coordinators**

In each participating clinic a liaison (program director or clinical supervisor) will be identified, to be responsible for coordinating training workshops and maintaining open communication with the study team regarding recruitment, data submission, technical assistance needs, and staff turnover and feedback.

**Data Safety and Monitoring Board**

An independent group of experts charged with reviewing study data for quality and integrity, adherence to the protocol, participant safety, study conduct and progress, quality of data analysis and interpretation, and making determinations regarding study continuation, modifications, and if needed study suspensions/terminations will be appointed. The DSMB will be independent from, but will work in conjunction with, the PI and IRB. The DSMB will meet annually and be empowered to request protocol edits and to suspend or terminate the study if indicated by interim results. The PI is responsible for immediately reporting adverse events and other unintended effects of study interventions or study conduct to the sponsoring agency, IRB, and DSMB.
