# Supplementary material for: Family Support Protocol for Adolescent Internalizing Disorders: Protocol for a Pre-Post Quantitative Treatment Development Study
Source: JMIR Res Protoc. 2024 Sep 16;13:e64332. doi: 10.2196/64332 (PMC11443177; doi:10.2196/64332)
Supplement: Multimedia Appendix 4 [file resprot_v13i1e64332_app4.pdf]

SUMMARY STATEMENT
(Privileged Communication)

PROGRAM CONTACT:
CARRIE Mulford
301-827-6473
carrie.mulford@nih.gov

Release Date: **07/13/2023**
Revised Date: **07/13/2023**

Application Number: **1R34DA056026-01A1**
Formerly: **1R34DA056026-01**

Principal Investigator

HOGUE, AARON

Applicant Organization: PARTNERSHIP TO END ADDICTION

Review Group: **IPTA**
Interventions to Prevent and Treat Addictions Study Section

Meeting Date: **06/22/2023**
Council: **OCT 2023**
Requested Start: **02/01/2024**
Opportunity Number: **PAR-22-183**
PCC: **CM/CFM**
Dual IC(s): **AA**

Project Title: **Testing the Feasibility of a Family-based Adjunctive Treatment Protocol for Targeting Co-Occurring Internalizing Disorders among Adolescents with SUD**
SRG Action: **Impact Score:30**
Next Steps: **Visit https://grants.nih.gov/grants/next_steps.htm**
Human Subjects: 30-Human subjects involved - Certified, no SRG concerns
Animal Subjects: 10-No live vertebrate animals involved for competing appl.
Gender: 1A-Both genders, scientifically acceptable
Minority: 1A-Minorities and non-minorities, scientifically acceptable
Age: 6A-Children and Adults, scientifically acceptable

Project Year	Direct Costs Requested	Estimated Total Cost
1	150,000	243,390
2	150,000	243,390
3	150,000	243,390
TOTAL	450,000	730,171

ADMINISTRATIVE BUDGET NOTE: The budget shown is the requested budget and has not been adjusted to reflect any recommendations made by reviewers. If an award is planned, the costs will be calculated by Institute grants management staff based on the recommendations outlined below in the COMMITTEE BUDGET RECOMMENDATIONS section.

1R34DA056026-01A1 Hogue, Aaron

RESUME AND SUMMARY OF DISCUSSION: This resubmitted application proposes to evaluate the acceptability and fidelity benchmarks of the Family Support Protocol for Adolescent Internalizing Disorders (Fam-AID), and examine FAM-AID's outcomes on family member session attendance and adolescent internalizing disorder symptoms. During discussion, the panel noted the study's significance in addressing important gaps in clinical treatment of comorbid substance use and internalizing disorders among adolescents through a culturally responsive and flexible intervention that has the potential to address scalability barriers, increase sustainability efforts, and improve adolescent outcomes. Leveraging the importance of family members and caregivers in treatment was considered innovative. However, the content of the Fam-AID was not considered particularly innovative. The investigative team is excellent with requisite and complementary expertise to conduct the project. Additional strengths noted underscore the rigor of the study design, including use of Interrupted Time Series; established community partnerships; and responsiveness to previous critiques. A few weaknesses related to the approach were also identified, including lack of clarity and justification on the eligibility criteria that require a youth to live with a caregiver who can attend the treatment sessions; insufficient justification for not requiring participating caregivers to attend all sessions, which may lead to between-subject variation in caregiver participation; and lack of attention and consideration to potential content overlap between the treatment and treatment-as-usual conditions. Following the discussion, the reviewers concluded that the application's strengths outweigh its addressable weaknesses and the project's potential impact to inform and augment treatment of co-occurring substance use and internalizing disorders among adolescents is high.

DESCRIPTION (provided by applicant): This R34 will develop and test an adjunctive treatment protocol for addressing co-occurring internalizing disorders among adolescents (age 13-21) enrolled in usual care for substance use problems. Internalizing disorders (IDs), primarily depression and anxiety, are highly prevalent among youth receiving community-based treatment for substance use disorders (SUDs), and unresolved ID issues significantly interfere with SUD treatment and recovery processes. Yet, the youth SUD clinical workforce is not systematically educated or trained in evidence-based practices for ID; thus, line services for youth SUD do not systematically target IDs. The research literature offers a few integrated behavioral models for simultaneously treating both SUDs and IDs in youth; however, such models feature intensive manualized procedures that have proven cumbersome to scale and deliver in frontline settings. As a result, the clinical workforce, though desiring ID-focused training, currently has inadequate resources for treating IDs effectively. A promising solution to reduce this quality gap is developing a modular, family-based protocol to augment routine care for comorbid SUD/ID by directly targeting ID as a primary treatment goal: Family Support Protocol for Adolescent Internalizing Disorders (Fam-AID). Fam-AID will contain six modules designed to enhance existing SUD services and to be delivered in any sequence to meet client needs: (1) Family Engagement of caregivers in treatment planning and services; (2) Relational Reframing of family constraints, resiliencies, and social capital connected to the youth's ID symptoms; (3) Functional Analysis of the youth's ID symptoms and related behaviors; (4) Core cognitive-behavioral treatment techniques to address the youth's ID symptoms and functional needs, featuring transdiagnostic interventions (emotion acceptance, emotional exposure, behavioral activation) to address negative affect and emotional dysregulation underlying both depression and anxiety; (5) Family Psychoeducation and Safety Planning focused on education about comorbid SUD/ID and prevention of youth self-harm. The study will follow the NIDA blueprint for Stage 1 development of behavioral protocols. In the Pilot Stage we will use rapid cycle prototyping methods in collaboration with end-user stakeholders at two Pilot sites to: (1) Draft protocol delivery and fidelity guidelines adapted from existing study team resources; (2) Solicit clinic staff and client input on protocol content and delivery via cognitive interviewing; (3) Pilot the prototype components on 2-3 cases at each site. We will then conduct an Interrupted Time Series

Study for N = 60 comorbid SUD/ID cases across two sites serving diverse youth: 30 will receive TAU (historical control), and then following line staff training in the protocol, 30 new cases will receive TAU enhanced by Fam- AID. Aim 1 will examine Fam-AID cases for protocol acceptability via therapist and client interviews and fidelity benchmarks via therapist- and observer-report protocol fidelity data. Aim 2 will compare Fam-AID versus TAU for immediate outcomes on family attendance in sessions and ultimate outcomes on youth ID symptoms at Baseline, 3-, and 6-month follow-up.

PUBLIC HEALTH RELEVANCE: Internalizing disorders, primarily anxiety and depression, are highly prevalent among adolescents receiving community-based treatment for SUD; moreover, unresolved internalizing issues significantly interfere with adolescent SUD treatment and recovery. Yet, routine services for youth SUD do not systematically target internalizing disorders. The proposed study will address this quality gap by developing a modular, family-based protocol to augment routine youth SUD care by directly targeting co-occurring internalizing problems as a primary treatment goal: Family Support Protocol for Adolescent Internalizing Disorders (Fam-AID).

CRITIQUE 1

Significance: 3
Investigator(s): 1
Innovation: 1
Approach: 3
Environment: 1

Overall Impact: This R34 resubmission seeks to test an adjunctive, modular protocol (Family Support Protocol for Adolescent Internalizing Disorders; FAM-AID) to treat internalizing disorders among youth ages 13 to 17 enrolled in usual care for substance use problems. The proposal consists of an initial pilot phase (n = 6) followed by an interrupted times series design including 60 youth comorbid in substance use and internalizing disorders (with 30 receiving treatment as usual, followed by 30 receiving FAM-AID). The proposal's first aim is to evaluate FAM-AID's feasibility and the second aim is to test the impact of FAM-AID (relative to the treatment as usual control) on participation/session attendance as well as internalizing disorder outcomes at 3- and 6-month follow-ups. This innovative project is positioned to have a high impact. The investigators are excellent and have the necessary expertise to carry out the proposed work. The protocol tested in this proposal has the potential to be an effective, flexible, and scalable approach for addressing comorbid internalizing disorders among youth engaged in substance use treatment, which is significant given that internalizing disorders are highly comorbid with substance use but often go unaddressed in substance use treatment. On balance, the strengths of the proposal far outweigh some modest weaknesses, which include the protocol not being applicable to youth with caregivers who are unwilling or unable to participate as well as some question as to the viability of the available participant pool.

1. Significance:

Strengths

- SUD and Internalizing Disorders (ID) are highly comorbid among adolescents.
- On account of this comorbidity, there is a clear need for interventions that target both treatment of SUD and ID, because the presence of ID can disrupt the effects of SUD treatment.
- There are available, manualized integrated treatment models for SUD and ID, but because of their cumbersome rigidity and structure, they have proven burdensome for clinicians to deliver and have proven difficult to evaluate.

- The proposed intervention seeks to overcome these scalability barriers of integrated treatments by using an adjunctive model with modularized core elements.
- The defined but yet to-be-developed intervention modules will utilize evidence-based approaches.
- Supportive of scalability and uptake, evidence from clinical surveys suggests that SUD-focused clinicians are interested in, motivated to, and capable of (i.e., they have the person hours) integrating ID treatment into their SUD treatment.

Weaknesses

- While making the adjunctive, modular treatment for internalizing disorders family-based makes great sense and is theoretically and empirically justified, it does limit the applicability of the protocol to those youth with comorbid SU and ID who have caregivers who are willing (and able) to participate and engage in a multi-session treatment program. This limitation has some weight since youth with comorbid SU and ID are more likely to come from at-risk backgrounds characterized by single-parent, low-income households, where parental participation in a protocol such as this might be more difficult or challenging.

2. Investigator(s):

Strengths

- The investigative team (PI Hogue, Co-I Ehrenreich-May, and consultants Henderson, Southam-Gerow, Diamond, and Jensen-Doss) have the necessary expertise to carry out all of the proposed work.

Weaknesses

- None.

3. Innovation:

Strengths

- The modular and adjunctive design maximizes flexibility and adaptability as well as facilitates integration with existing SUD treatment protocols.
- Given the importance of families to recovery and treatment of both SUDs and IDs, the incorporation of family and caregivers via evidence-based practices into the treatment is a novel and important aspect of the proposal.

Weaknesses

- None.

4. Approach:

Strengths

- The use of an interrupted time series design is a unique but appropriate approach for this proposal, and the merits of its application here is well described and justified.
- The PI has prior experience developing and piloting adjunctive interventions.
- Although exploratory, biological sex will be included as a moderator.

- Partnering sites are well suited for this effort and relationships between the PI and these partnering sites are well established, as verified via letters of support.
- Procedures for all the initial pilot phase and the subsequent interrupted time series phase are well-described and comprehensive.
- The pilot phase will seek feedback from all stakeholders: clinicians, families, and youth.
- There is a sustained focus on making sure the intervention is culturally attuned, adaptive, and flexible.
- The components and content of the intervention are built from empirically tested strategies for treating internalizing disorders.

Weaknesses

- The proposal indicates that the performance sites should provide a sufficient pool of participants for the purposes of this proposal. However, one factor not taken into account in these calculations is the proportion of youth who will be excluded because they do not have a caregiver who is willing or able to participate. Even with this taken into consideration, there likely is still a sufficient number of youth and corresponding caregivers to meet the sample objectives for the pilot phase and ITS phase. But ideally the proposal would have provided an estimate (and justification for this estimate) for the proportion of youth who will not be eligible due to their having a caregiver who is unwilling or unable to participate.
- Although having a caregiver that the youth lives with who can attend is an eligibility criteria, it is not required that this caregiver participate in all sessions. This will likely lead to substantial between-subject variation in the amount of caregiver participation. Because caregiver participation is not included as a control, it is a potential unaccounted for confound.

5. Environment:

Strengths

- The environments at the partnering institutions for this proposal (Partnership to end addiction, University of Miami) as well as the three testing/performance sites are excellent for the proposed research.

Weaknesses

- None.

Study Timeline:

Strengths

- Proposed timeline is reasonable.

Weaknesses

- None.

Protections for Human Subjects:

Acceptable Risks and/or Adequate Protections

- Acceptable.

Data and Safety Monitoring Plan (Applicable for Clinical Trials Only):

Acceptable

Inclusion Plans:

- Sex/Gender: Distribution justified scientifically
- Race/Ethnicity: Distribution justified scientifically
- For NIH-Defined Phase III trials, Plans for valid design and analysis: Not applicable
- Inclusion/Exclusion Based on Age: Distribution justified scientifically

Vertebrate Animals:
Not Applicable (No Vertebrate Animals)

Biohazards:
Not Applicable (No Biohazards)

- NA

Resubmission:

- The modifications to the proposal adequately addressed the major concerns raised by previous reviewers.

Applications from Foreign Organizations:

- NA

Select Agents:

- NA

Resource Sharing Plans:
Acceptable

Authentication of Key Biological and/or Chemical Resources:
Not Applicable (No Relevant Resources)

Budget and Period of Support:
Recommend as Requested

CRITIQUE 2

Significance: 2
Investigator(s): 2
Innovation: 3

Approach: 3
Environment: 2

Overall Impact: This application proposes to develop and test an adjunctive treatment protocol for addressing co-occurring internalizing disorders among adolescents (age 13-17) enrolled in treatment for substance use problems in community settings. Internalizing disorders (IDs), primarily depression and anxiety, are prevalent among youth receiving community-based treatment for substance use disorders (SUDs), and yet they are rarely addressed directly in substance use treatment. This study proposes an interrupted time series design across 2 community clinics, where providers will be trained in the model and families will be assigned to usual care (N=30) or usual care with the FAM-AID intervention (N=30) after training of providers. Aim 1 will focus on feasibility and Aim 2 will test outcomes on depression, anxiety, and substance use. This application builds on the work of the investigator who has multiple grants focused on substance use prevention with this population. This application has a number of strengths, including a unique design and strong community partnerships. Weaknesses center around disentangling the control group from the intervention group, as there is likely a lot of overlap in content between these 2 groups of participants.

1. Significance:

Strengths

- Depression and substance use disorders co-occur with rates as high as 70% overlap.
- Presence of internalizing disorders significantly disrupts recovery in adolescents with substance use.
- Addiction specialists rarely have clinical training focused on addressing mental health concerns.
- Self-regulation difficulties are core to both the development of internalizing disorders and substance use.

Weaknesses

- None noted by reviewer.

2. Investigator(s):

Strengths

- Dr. Aaron Hogue is the Vice President and Director of the Family Adolescent and Clinical Technology and Science at PEA.
- He is a senior researcher with a history of funded research in the area of addiction and prevention of substance abuse with adolescents.
- Other members of the team complement his expertise in treatment of internalizing disorders and statistical analyses.

Weaknesses

- None noted by reviewer.

3. Innovation:

Strengths

- The innovation of FAM-AID is discussed as an evidenced based intervention that is flexibly delivered.
- FAM-AID directly targets families in the treatment of SUD, which is less common in community settings.
- FAM-AID integrates both a CBT and family therapy approach to treatment.

Weaknesses

- The content of FAM-AID is not particularly innovative, and is consistent with evidence-based approaches for internalizing disorders.

4. Approach:

Strengths

- The research plan begins with a pilot phase, where materials will be assembled and feedback from providers will be collected.
- The research design is an interrupted time series, where families are assigned to condition in phase one with TAU, and then providers will be trained and the next wave of families will receive TAU plus FAM-AID.
- FAM-AID content includes a range of evidence-based content, including CBT techniques, educational content and planning, functional analysis of ID symptoms, and cultural adaptations for each family.
- Youth ages 13-17 will be recruited from community partners. Youth will have both SUD and ID at time of recruitment.
- Assessments will be conducted at baseline, 3 months, and 6 months and include measures of emotional regulation and ID.

Weaknesses

- Although the application makes a strong case for SUD treatment being distinct from treatments of ID, there is likely content overlap in the TAU and treatment conditions given the broad nature of this content. Little information about TAU is included in this application.
- It is unclear if the assessments are both youth and parent report. It is assumed that both youth and parent will report on symptoms.

5. Environment:

Strengths

- The Partnership to End Addiction is a national non-profit research and public policy organization housed in New York City. The organization appears to house multiple federal grants and resources for communities focused on substance use prevention and addiction services. The organization conducts outreach and collaborates with community settings, which is a strength for supporting this type of research.

Weaknesses

- None noted by reviewer.

Study Timeline:

Strengths

- The timeline is reasonable for this application.

Weaknesses

- None noted by reviewer.

Protections for Human Subjects:
Acceptable Risks and/or Adequate Protections
Data and Safety Monitoring Plan (Applicable for Clinical Trials Only):
Acceptable.

Inclusion Plans:

- Sex/Gender: Distribution justified scientifically
- Race/Ethnicity: Distribution justified scientifically
- For NIH-Defined Phase III trials, Plans for valid design and analysis:
- Inclusion/Exclusion Based on Age: Distribution justified scientifically

Vertebrate Animals:
Not Applicable (No Vertebrate Animals)

Biohazards:
Not Applicable (No Biohazards)

Resubmission:

- The application was responsive to prior reviews.

Applications from Foreign Organizations:
Not Applicable (No Foreign Organizations)

Select Agents:
Not Applicable (No Select Agents)

Resource Sharing Plans:
Acceptable

Authentication of Key Biological and/or Chemical Resources:
Not Applicable (No Relevant Resources)

Budget and Period of Support:
Recommend as Requested

CRITIQUE 3

Significance: 2
Investigator(s): 1
Innovation: 3
Approach: 3
Environment: 1

Overall Impact: The proposed study aims to develop a promising solution to address the significant gap in the treatment of adolescents with SUDs and co-occurring IDs. Using a modular, family-based protocol to augment routine care for comorbid SUD/ID by directly targeting ID as a primary treatment goal, the Family Support Protocol for Adolescent Internalizing Disorders (Fam-AID) will contain five modules designed to enhance existing SUD services and to be delivered in any sequence to meet client needs. Overall, the flexible modular adjunct treatment approach is both significant and innovative, the approach is rigorous, and the team has a strong record of productivity and expertise in this area, enhancing feasibility. Some minor weaknesses are noted in the approach.

1. Significance:

Strengths

- The proposed study addresses a significant gap in the treatment of adolescents with SUDs and co-occurring IDs, who make up a sizable proportion of youth in community-based treatment. Comorbid IDs can significantly disrupt SUD treatment and recovery. While clinicians tend to lack systematic training in evidence-based practices in IDs, the proposed adjunctive modular Fam-AID protocol targeting IDs can improve effectiveness for comorbid SUD and ID.
- Studies surveying SUD clinicians indicate that training and protocols for treating co-occurring ID in youth are uniformly needed. Existing integrated behavioral models have intensive manualized procedures that are difficult to scale.
- Rather than directly targeting SUD outcomes, the Fam-AID protocol is designed as an adjunctive to complement whatever current interventions are being carried out by a clinician; therefore, clinicians would not need to significantly change their existing practices. This increases the potential sustainability of the model if it is deemed successful.

Weaknesses

- None noted.

2. Investigator(s):

Strengths

- This is a highly competent team with complementary skills and experience. The PI is an established researcher with an extensive record of funding and experience on SUD among adolescents, RCTs, and clinical workforce training studies.

Weaknesses

- None noted.

3. Innovation:

Strengths

- The use of family engagement techniques that are evidence-based is innovative as family members are not typically centralized in frontline SUD services for youth.
- The ability of therapists and clients to customize and collaboratively select which CBT techniques to use in addressing ID is novel. By allowing flexibility in the delivery sequence, the intervention can meet the tailored needs of individual clients.

Weaknesses

- While the adjunctive intervention is novel, the elements that comprise it are core characteristics of existing evidence-based treatment.

4. Approach:

Strengths

- The modules used in the approach are rigorous and based on core elements of evidence-based treatment. The flexibility afforded in the model can increase implementation.
- The investigative team's experience in developing and testing interventions enhances feasibility of the proposed study.
- The Interrupted Time Series model is a strength and well-conceived, and the approach justifies the study design rationale while addressing alternative designs.
- Fidelity procedures for protocol coverage and dose are described.
- The data collection will address barriers to family engagement and generate strategies to solve or ameliorate barriers, enhance family motivation to participate, and bolster site policies to facilitate family involvement.

Weaknesses

- It is not entirely clear how many staff interviews will be conducted in Phase 1 and the number of individuals (staff and clients) interviewed during this phase do not appear to be included in the planned inclusion enrollment report.
- It was not clear why it was requirement for the youth to live with a primary caregiver who can attend treatment sessions, although it was noted that this was a criterion the PI has used in two prior RCTs of family-focused models.
- Minor: The Introduction and Revised Strategy list 13-17 as the target age but the abstract, Study Population Characteristics (p.87), and Inclusion of Individuals Across the Lifespan (p.88) state the age range is 13-21. Likewise, the Introduction states that references to "mindfulness" have been replaced with "emotion acceptance" but these are still included in the Protocol Synopsis. Also, the Study Population Section needs to be updated to include the additional criterion of living with a caregiver. Finally there is reference to six modules in the abstract and protocol synopsis even though only five modules will be used.

5. Environment:

Strengths

- The collaborating institutions provide a supportive research environment within which to conduct the proposed study.

Weaknesses
- None noted.

Study Timeline:
Strengths
- Timeline is feasible and well justified.

Weaknesses
- None noted.

Protections for Human Subjects:
Acceptable Risks and/or Adequate Protections
- Potential risks and protections adequately described.

Data and Safety Monitoring Plan (Applicable for Clinical Trials Only):
Acceptable
- o Plan is acceptable.

Inclusion Plans:
- Sex/Gender: Distribution justified scientifically
- Race/Ethnicity: Distribution justified scientifically
- For NIH-Defined Phase III trials, Plans for valid design and analysis: Scientifically acceptable
- Inclusion/Exclusion Based on Age: Distribution justified scientifically
- Acceptable.

Vertebrate Animals:
Not Applicable (No Vertebrate Animals)

Biohazards:
Not Applicable (No Biohazards)

Resubmission:
- The application is highly responsive to the reviews.

Applications from Foreign Organizations:
Not Applicable (No Foreign Organizations)

Select Agents:
Not Applicable (No Select Agents)

Resource Sharing Plans:

Acceptable

Authentication of Key Biological and/or Chemical Resources:

Not Applicable (No Relevant Resources)

Budget and Period of Support:

Recommend as Requested

THE FOLLOWING SECTIONS WERE PREPARED BY THE SCIENTIFIC REVIEW OFFICER TO SUMMARIZE THE OUTCOME OF DISCUSSIONS OF THE REVIEW COMMITTEE, OR REVIEWERS' WRITTEN CRITIQUES, ON THE FOLLOWING ISSUES:

PROTECTION OF HUMAN SUBJECTS: ACCEPTABLE

INCLUSION OF WOMEN PLAN: ACCEPTABLE

INCLUSION OF MINORITIES PLAN: ACCEPTABLE

INCLUSION ACROSS THE LIFESPAN: ACCEPTABLE

COMMITTEE BUDGET RECOMMENDATIONS: The budget was recommended as requested.

Footnotes for 1R34DA056026-01A1; PI Name: Hogue, Aaron

NIH has modified its policy regarding the receipt of resubmissions (amended applications).See Guide Notice NOT-OD-18-197 at https://grants.nih.gov/grants/guide/notice-files/NOT-OD-18-197.html. The impact/priority score is calculated after discussion of an application by averaging the overall scores (1-9) given by all voting reviewers on the committee and multiplying by 10. The criterion scores are submitted prior to the meeting by the individual reviewers assigned to an application, and are not discussed specifically at the review meeting or calculated into the overall impact score. Some applications also receive a percentile ranking. For details on the review process, see http://grants.nih.gov/grants/peer_review_process.htm#scoring.

MEETING ROSTER

Interventions to Prevent and Treat Addictions Study Section
Risk, Prevention and Health Behavior Integrated Review Group
CENTER FOR SCIENTIFIC REVIEW
IPTA
06/22/2023 - 06/23/2023

Notice of NIH Policy to All Applicants: Meeting rosters are provided for information purposes only. Applicant investigators and institutional officials must not communicate directly with study section members about an application before or after the review. Failure to observe this policy will create a serious breach of integrity in the peer review process, and may lead to actions outlined in NOT-OD-22-044 at https://grants.nih.gov/grants/guide/notice-files/NOT-OD-22-044.html, including removal of the application from immediate review.

CHAIRPERSON(S)

PIPER, MEGAN E, PHD
PROFESSOR
DEPARTMENT OF MEDICINE
UNIVERSITY OF WISCONSIN-MADISON
MADISON, WI 53711

MEMBERS

ALETRARIS, LYDIA, PHD *
ASSOCIATE RESEARCH SCIENTIST
SCHOOL OF SOCIAL WORK
UNIVERSITY OF GEORGIA
ATHENS, GA 30602

BERNSTEIN, STEVEN L, MD
PROFESSOR
DEPARTMENT OF EMERGENCY MEDICINE
GEISEL SCHOOL OF MEDICINE AT DARTMOUTH
LEBANON, NH 03756

BINSWANGER, INGRID A, MD, MPH
SENIOR CLINICIAN INVESTIGATOR
INSTITUTE FOR HEALTH RESEARCH
KAISER PERMANENTE
AURORA, CO 80014

BRICKER, JONATHAN B, PHD
PROFESSOR
DIVISION OF PUBLIC HEALTH SCIENCES
FRED HUTCHINSON CANCER RESEARCH CENTER
UNIVERSITY OF WASHINGTON
SEATTLE, WA 98109

CLAUS, ERIC D, PHD
ASSOCIATE PROFESSOR
DEPARTMENT OF BIOBEHAVIORAL HEALTH
THE PENNSYLVANIA STATE UNIVERSITY
UNIVERSITY PARK, PA 16802

FENDRICH, MICHAEL, PHD *
SCIENTIFIC DIRECTOR
ADVOCATE AURORA RESEARCH INSTITUTE
MILWAUKEE, WI 53211

GAINES, TOMMI LYNN, DRPH *
ASSOCIATE PROFESSOR
DIVISION OF GLOBAL PUBLIC HEALTH
DEPARTMENT OF MEDICINE
SCHOOL OF MEDICINE
UNIVERSITY OF CALIFORNIA, SAN DIEGO
LA JOLLA, CA 92093

GARRISON, KATHLEEN A, PHD
ASSISTANT PROFESSOR
DEPARTMENT OF PSYCHIATRY
YALE SCHOOL OF MEDICINE
NEW HAVEN, CT 06510

GRYCZYNSKI, JAN, PHD
SENIOR RESEARCH SCIENTIST
FRIENDS RESEARCH INSTITUTE
BALTIMORE, MD 21201

HAGLE, HOLLY *
ASSOCIATE RESEARCH PROFESSOR
MISSOURI CENTER FOR ADDICTION RESEARCH
AND ENGAGEMENT
COLLEGE OF ARTS AND SCIENCE
UNIVERSITY OF MISSOURI
KANSAS CITY, MO 64110

JAGER, JUSTIN O, PHD *
ASSOCIATE PROFESSOR
COLLEGE OF HEALTH SOLUTIONS
THE SANFORD SCHOOL
ARIZONA STATE UNIVERSITY
TEMPE, AZ 85281

KELLEY, MICHELLE L, PHD *
PROFESSOR AND CHAIR
DEPARTMENT OF PSYCHOLOGY
COLLEGE OF SCIENCES
OLD DOMINION UNIVERSITY
NORFOLK, VA 23322

KOWALCHUK, ALICIA ANN, DO *
ASSOCIATE PROFESSOR
BAYLOR COLLEGE OF MEDICINE
HOUSTON, TX 77030

LANGDON, KIRSTEN JOHNSON, PHD *
ASSISTANT PROFESSOR
DEPARTMENT OF BEHAVIORAL AND SOCIAL SCIENCES
DEPARTMENT OF PSYCHIATRY AND HUMAN BEHAVIOR
BROWN UNIVERSITY
PROVIDENCE, RI 02912

LEDGERWOOD, DAVID M, PHD
PROFESSOR
DEPARTMENT OF PSYCHIATRY
AND BEHAVIORAL NEUROSCIENCES
SCHOOL OF MEDICINE
WAYNE STATE UNIVERSITY
DETROIT, MI 48201

MATHEW, AMANDA R, PHD *
ASSISTANT PROFESSOR
DEPARTMENT OF PREVENTIVE MEDICINE
RUSH UNIVERSITY
CHICAGO, IL 60612

MCHUGH, REBECCA KATHRYN, PHD
ASSOCIATE PROFESSOR
DEPARTMENT OF PSYCHIATRY
HARVARD MEDICAL SCHOOL
BELMONT, MA 02478

MCNEELY, JENNIFER, MD
ASSOCIATE PROFESSOR
DEPARTMENTS OF POPULATION HEALTH AND MEDICINE
SCHOOL OF MEDICINE
NEW YORK UNIVERSITY
NEW YORK, NY 10016

MCPHERSON, STERLING M, PHD
PROFESSOR AND DIRECTOR
PROGRAM OF EXCELLENCE IN ADDICTIONS RESEARCH
DEPARTMENT OF COMMUNITY AND BEHAVIORAL HEALTH
ELSON S. FLOYD COLLEGE OF MEDICINE
WASHINGTON STATE UNIVERSITY
SPOKANE, WA 99210

MILLS, SARAH, PHD, MPH *
ASSISTANT PROFESSOR
DEPARTMENT OF HEALTH BEHAVIOR
GILLINGS SCHOOL OF GLOBAL PUBLIC HEALTH
UNIVERSITY OF NORTH CAROLINA
CHAPEL HILL, NC 27599

MONTGOMERY, LATRICE, PHD
ASSOCIATE PROFESSOR
DEPARTMENT OF PSYCHIATRY AND
BEHAVIORAL NEUROSCIENCE
COLLEGE OF MEDICINE
UNIVERSITY OF CINCINNATI
CINCINNATI, OH 45229

MUMBA, MERCY N, PHD
ASSOCIATE PROFESSOR
CAPSTONE COLLEGE OF NURSING
THE UNIVERSITY OF ALABAMA
TUSCALOOSA, AL 35401

NAPPER, LUCY ELIZABETH, PHD *
ASSOCIATE PROFESSOR
DEPARTMENT OF PSYCHOLOGY
COLLEGE OF ARTS AND SCIENCES
LEHIGH UNIVERSITY
BETHLEHEM, PA 18015

POWELL, TERRINIEKA WILLIAMS, PHD *
ASSOCIATE PROFESSOR
POPULATION, FAMILY AND REPRODUCTIVE HEALTH
BLOOMBERG SCHOOL OF PUBLIC HEALTH
JOHNS HOPKINS UNIVERSITY
BALTIMORE, MD 21205

RAIFF, BETHANY R, PHD *
PROFESSOR
DEPARTMENT OF PSYCHOLOGY
COLLEGE OF SCIENCE AND MATHEMATICS
ROWAN UNIVERSITY
GLASSBORO, NJ 08028

SADASIVAM, RAJANI, PHD
PROFESSOR
DIVISION OF HEALTH AND IMPLEMENTATION SCIENCE
DEPARTMENT OF POPULATION AND QUANTITATIVE
HEALTH SCIENCES
UNIVERSITY OF MASSACHUSETTS MEDICAL SCHOOL
WORCESTER, MA 01605

SARAIYA, TANYA CHANDRESH, PHD *
ASSISTANT PROFESSOR
DEPARTMENT OF ALCOHOL & SUBSTANCE USE STUDIES
GRADUATE SCHOOL OF APPLIED AND PROFESSIONAL
PSYCHOLOGY
RUTGERS UNIVERSITY
PISCATAWAY, NJ 08854

SHEFFER, CHRISTINE ELIZABETH, PHD
PROFESSOR
DEPARTMENT OF HEALTH BEHAVIOR
ROSWELL PARK COMPREHENSIVE CANCER CENTER
BUFFALO, NY 14263

STATON, MICHELE, PHD
PROFESSOR
DEPARTMENT OF BEHAVIORAL SCIENCES
COLLEGE OF MEDICINE
UNIVERSITY OF KENTUCKY
LEXINGTON, KY 40536

STORMSHAK, ELIZABETH A, PHD
PROFESSOR
COLLEGE OF EDUCATION
PREVENTION SCIENCE INSTITUTE
UNIVERSITY OF OREGON
EUGENE, OR 97403

VILARDAGA, ROGER, PHD
ASSOCIATE PROFESSOR
DEPARTMENT OF PSYCHIATRY AND BEHAVIORAL SCIENCES
SCHOOL OF MEDICINE
DUKE UNIVERSITY
DURHAM, NC 27710

WATKINS, KATHERINE E, MD, MSHS *
SENIOR SCIENTIST
HEALTH CARE DIVISION
RAND CORPORATION
SANTA MONICA, CA 90407

WELSH, JUSTINE WITTENAUER, MD *
DIRECTOR OF THE EMORY HEALTHCARE ADDICTION SERVICES
DEPARTMENT OF PSYCHIATRY AND BEHAVIORAL SCIENCES
SCHOOL OF MEDICINE
EMORY UNIVERSITY
ATLANTA, GA 30307

YI, RICHARD, PHD
PROFESSOR AND DIRECTOR
DEPARTMENT OF PSYCHOLOGY
UNIVERSITY OF KANSAS
LAWRENCE, KS 66045

MAIL REVIEWER(S)

D'SOUZA, DEEPAK CYRIL, MD
PROFESSOR
DEPARTMENT OF PSYCHIATRY
YALE UNIVERSITY
WEST HAVEN, CT 06516

SCIENTIFIC REVIEW OFFICER

VIDAL, SARAH, PHD
SCIENTIFIC REVIEW OFFICER
CENTER FOR SCIENTIFIC REVIEW
NATIONAL INSTITUTES OF HEALTH
BETHESDA, MD 20892

EXTRAMURAL SUPPORT ASSISTANT

AMARE, MERON ERMIAS
LEAD EXTRAMURAL SUPPORT ASSISTANT
CENTER FOR SCIENTIFIC REVIEW
NATIONAL INSTITUTES OF HEALTH
BETHESDA, MD 20892

* Temporary Member. For grant applications, temporary members may participate in the entire meeting or may review only selected applications as needed.

Consultants are required to absent themselves from the room during the review of any application if their presence would constitute or appear to constitute a conflict of interest.
